# Supplementary material for: X-ray-diffraction and electrical-transport imaging of superconducting superhydride (La,Y)H10
Source: Nat Commun. 2025 Dec 18;16:11222. doi: 10.1038/s41467-025-66262-1 (PMC12714734; doi:10.1038/s41467-025-66262-1)
Supplement: Supplementary file 1 — Supplementary Information [file 41467_2025_66262_MOESM1_ESM.pdf]

SUPPLEMENTARY INFORMATION

X-ray Diffraction and Electrical Transport Imaging of Superconducting Superhydride (La,Y)H_{10}

Abdul Haseeb Manayil Marathamkottil[1*], Kui Wang[2], Nilesh P. Salke[2], Muhtar Ahart[2], Alexander C. Mark[2], Rostislav Hrubiak[3], Stella Chariton[4], Dean Smith[3], Vitali B. Prakapenka[4], Maddury Somayazulu[3], Nenad Velisavljevic[3,5], Russell J. Hemley[1,2,6*]

[1]*Department of Chemistry, University of Illinois Chicago, Chicago, IL 60607, USA,*

[2]*Department of Physics, University of Illinois Chicago, Chicago, IL 60607, USA,*

[3]*HPCAT, X-ray Science Division, Argonne National Laboratory, Lemont, IL 60439, USA,*

[4]*Center for Advanced Radiation Sources, University of Chicago, Chicago, IL 60637, USA,*

[5]*Physics Division, Lawrence Livermore National Laboratory, Livermore, CA 94550, USA,*

[6]*Department of Earth and Environmental Sciences, University of Illinois Chicago, Chicago, IL 60607, USA*

**E-mail: amanay2@uic.edu, rhemley@uic.edu*

This Supplementary Information provides additional structural, transport, and characterization data supporting the main text. A full list of figures, tables, and notes is provided below.

Table of Contents

Figures

1. **Figure S1**. XRD patterns and refinement of coexisting $(La,Y)H_{10}$ phases at 168 GPa.
2. **Figure S2**. Comparison of $La_{0.9}Y_{0.1}$ samples at 158 and 172 GPa
3. **Figure S3**. DAC assembly and optical images before and after laser heating.
4. **Figure S4**. P – V comparison of La, $La_{0.9}Y_{0.1}$, LaH_{10}, and $(La,Y)H_{10}$ phases.
5. **Figure S5**. XRD collection positions for $(La,Y)H_{10}$ at 153 and 136 GPa.
6. **Figure S6**. Synchrotron XRD patterns and refinements of $(La,Y)H_{10}$ at 136 GPa.
7. **Figure S7**. XRD patterns confirming cubic and hexagonal phases at 153 and 136 GPa.
8. **Figure S8**. Spatially resolved XRD analysis of the cubic phase at 136 GPa.
9. **Figure S9**. XDI maps with local pressure variation at 153 GPa.
10. **Figure S10**. Temperature-dependent resistance of $(La,Y)H_{10}$ at 153 GPa.
11. **Figure S11**. Current–voltage characteristics of $(La_{0.9}Y_{0.1})H_{10}$ at 146 and 136 GPa.
12. **Figure S12**. Temperature-dependent four-probe partial resistance traces at 142 GPa.
13. **Figure S13**. Temperature-dependent four-probe partial resistance traces at 136 GPa.
14. **Figure S14**. SEM-EDS characterization of $La_{0.9}Y_{0.1}$ alloy.
15. **Figure S15**. Synchrotron XRD patterns of $La_{0.9}Y_{0.1}$ alloy at ambient conditions.
16. **Figure S16**. Diamond-edge Raman spectra for pressure calibration

Tables

1. **Table S1**. Results of SEM–EDS analysis for $La_{0.9}Y_{0.1}$ alloy

Note

1. **Note 1**. Volume-Based Stoichiometry Analysis of $(La,Y)H_{10}$

References

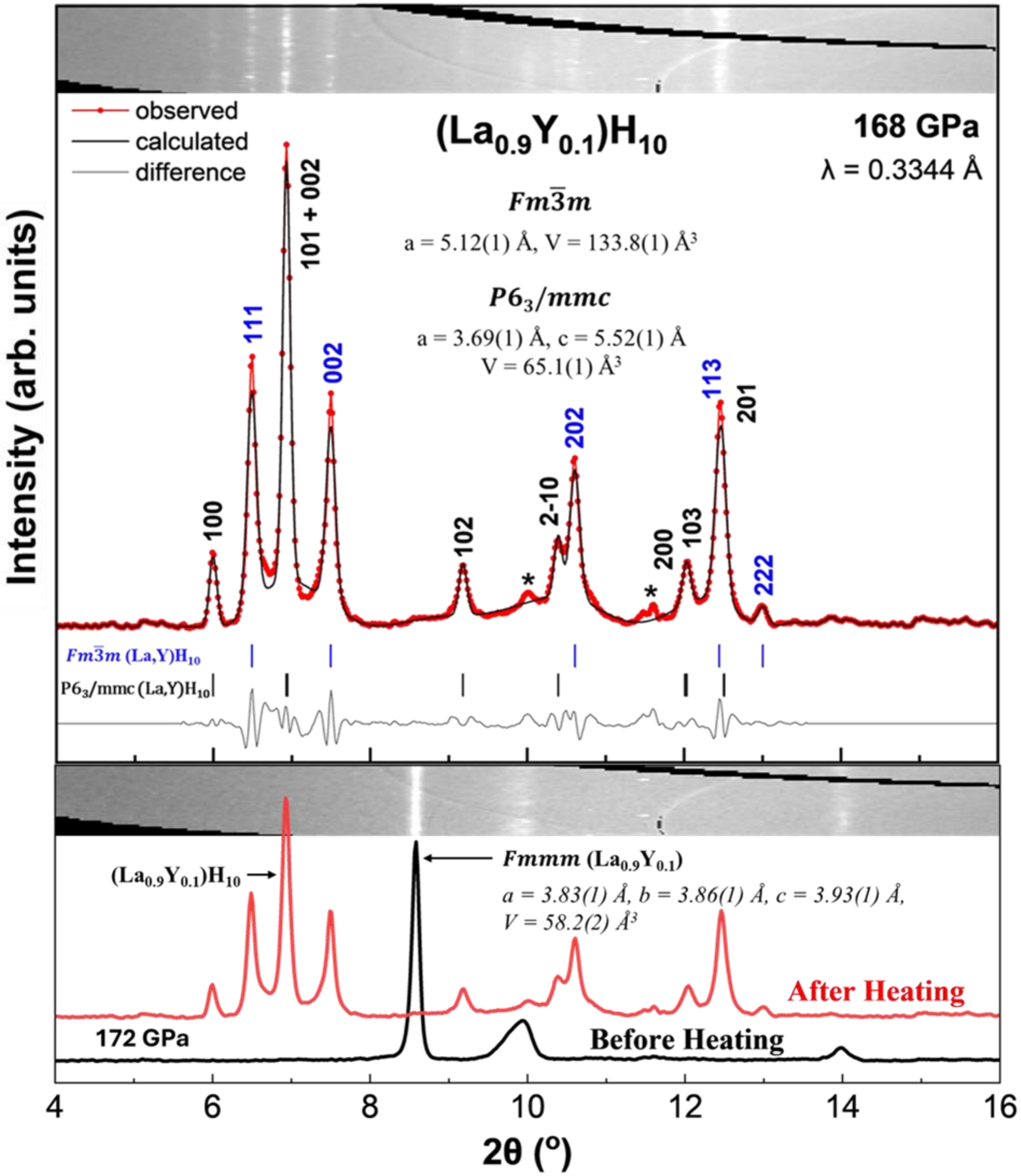

Figure S1: XRD patterns and structural refinement of coexisting (La,Y)H_{10} phases at 168 GPa. **Top**: Experimental synchrotron XRD patterns and Le Bail refinements of the $Fm\bar{3}m$ and $P6_3/mmc$ (La,Y)H_{10} phases at 168 GPa. The experimental data, fit, and residuals are shown in red, black, and gray, respectively. Refined lattice parameters for both phases are indicated. **Bottom**: Experimental XRD patterns of $La_{0.9}Y_{0.1}$ at 172 GPa before and after laser heating. The pre-heating pattern corresponds to the distorted-cubic $Fmmm$ phase. The post-heating pattern shows the formation of (La,Y)H_{10}, with the top refinement corresponding to the (La,Y)H_{10} phases. The peaks marked with "*" correspond to unidentified or sample-environment contributions. Insets show representative 2D diffraction images for reference.

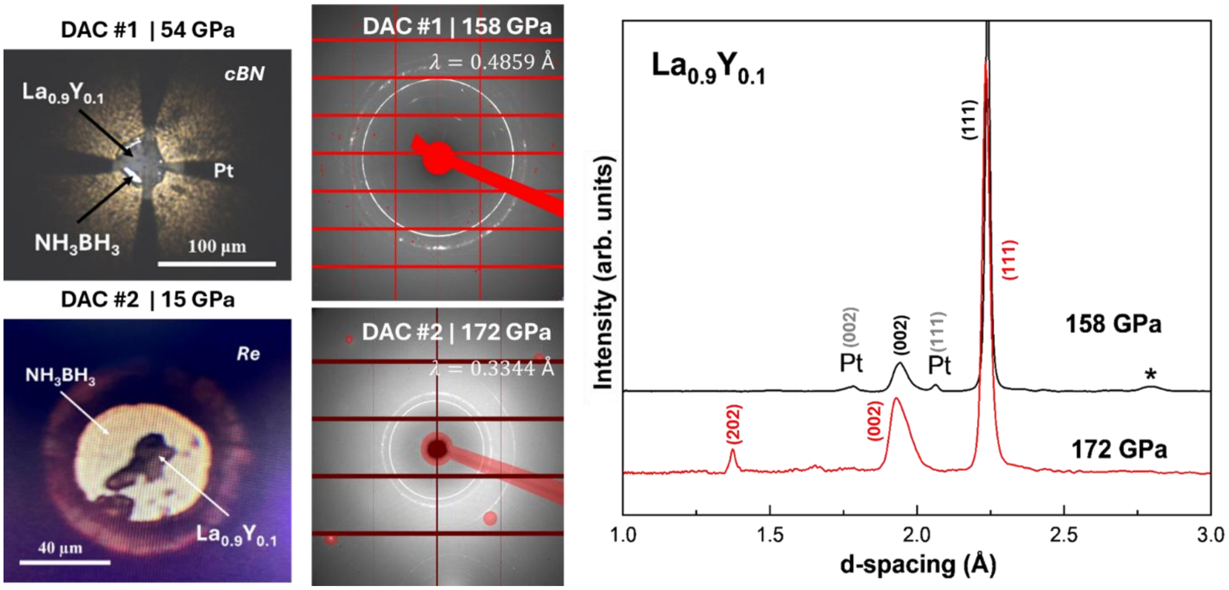

Figure S2: Comparison of $La_{0.9}Y_{0.1}$ samples at 158 and 172 GPa. **Left**: Optical images of the loaded chambers in DAC #1 and DAC #2, illustrating differences in sample environment. **Center**: 2D diffraction images, showing spotty rings at 158 GPa and uniform powder rings at 172 GPa. **Right**: Integrated XRD patterns. At 158 GPa (DAC #1), additional reflections from the cell assembly are present: peaks at d ≈ 1.79 and 2.2 Å correspond to fcc-Pt, while broad features at d ≈ 2.80 and 4.0 Å are marked as impurity and likely arise from amorphous gasket contributions. At 172 GPa (DAC #2), the pattern is cleaner and dominated by La–Y reflections.

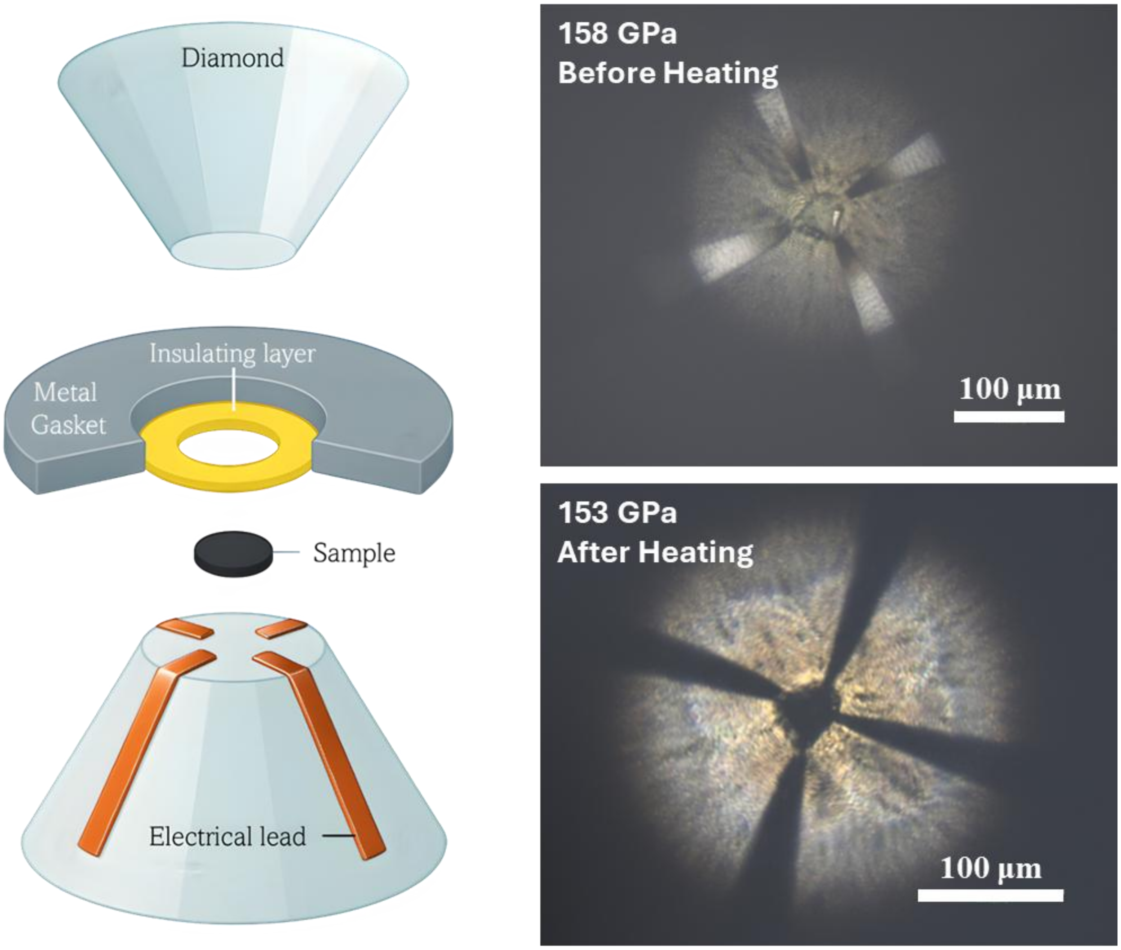

Figure S3: Left: Schematic illustration of the DAC assembly configured for four-probe electrical transport measurements. **Right**: Optical images of the (La,Y)H_{10} DAC #1 sample at 158 GPa before laser heating (transmitted and reflected light) and after heating at 153 GPa (transmitted light). A clear volume expansion is observed following synthesis, as evidenced by the transition of the initially transparent ammonia borane region to an opaque state on the right side of the culet.

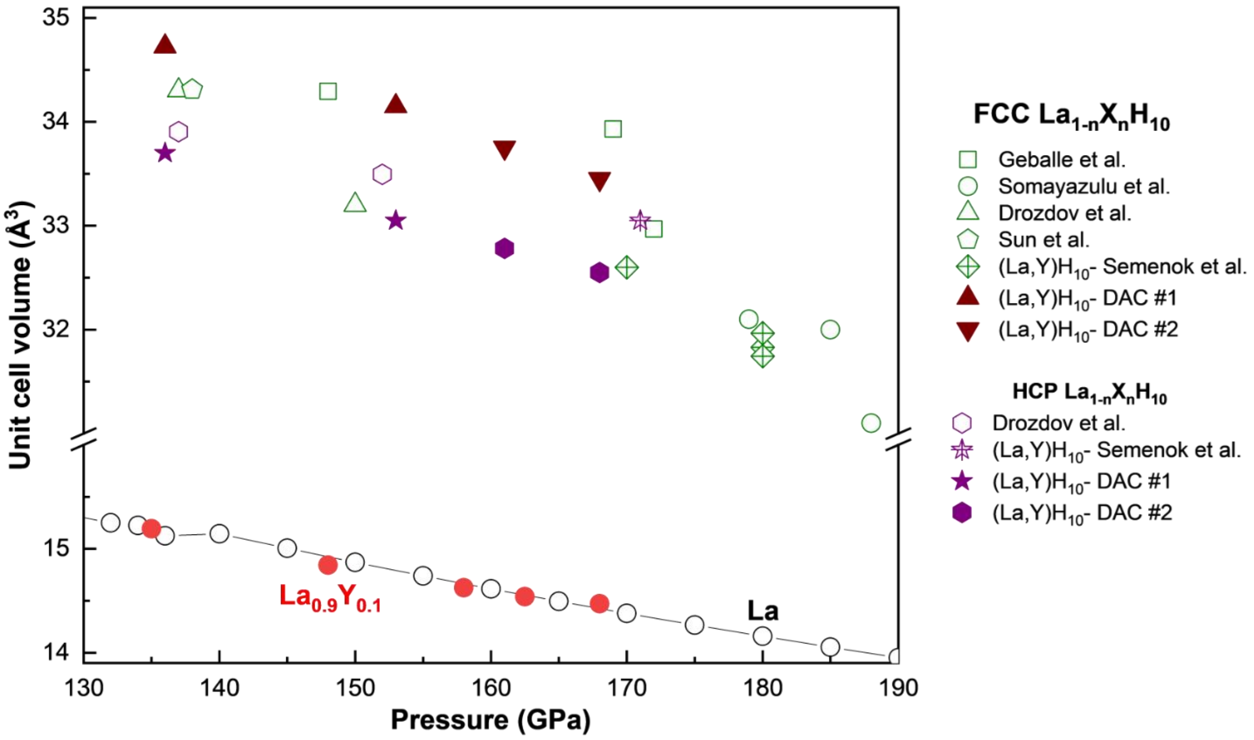

Figure S4: Pressure–unit cell volume comparison of La, $La_{0.9}Y_{0.1}$, LaH_{10}, and $(La,Y)H_{10}$ phases. The figure shows experimental data collected in this study and from previous literature[1–5]. Filled symbols represent data from this work, while open symbols correspond to literature values. Pressure calibration for our measurements was performed using the diamond Raman edge method. Some literature data were calibrated using the hydrogen vibron[3,4], which may underestimate the true pressure, leading to actual pressures being higher than reported.

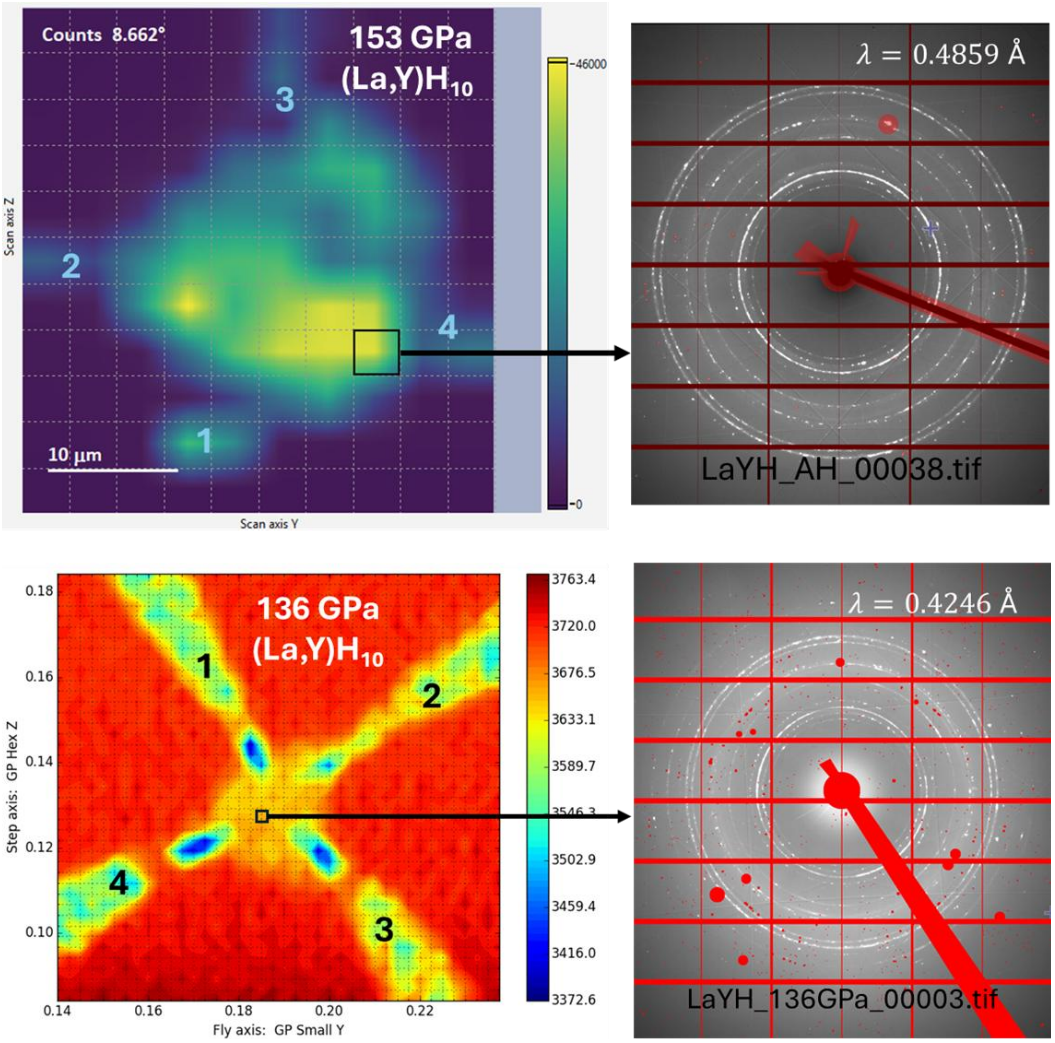

Figure S5: XRD collection positions for (La,Y)H_{10} at 153 GPa and 136 GPa in DAC #1, corresponding to the patterns shown in Fig. 1 and Fig. S5. **Left**: XDI map of the sample chamber at 153 GPa and 2D scan of the chamber at 136 GPa, with electrodes and sampling locations marked for spatial reference. **Right**: representative 2D diffraction images collected at the indicated locations. The 153 GPa pattern was collected near the electrode region, where Pt peaks are visible, while the 136 GPa pattern was collected at the sample center, where Pt contributions are within the signal-to-noise ratio.

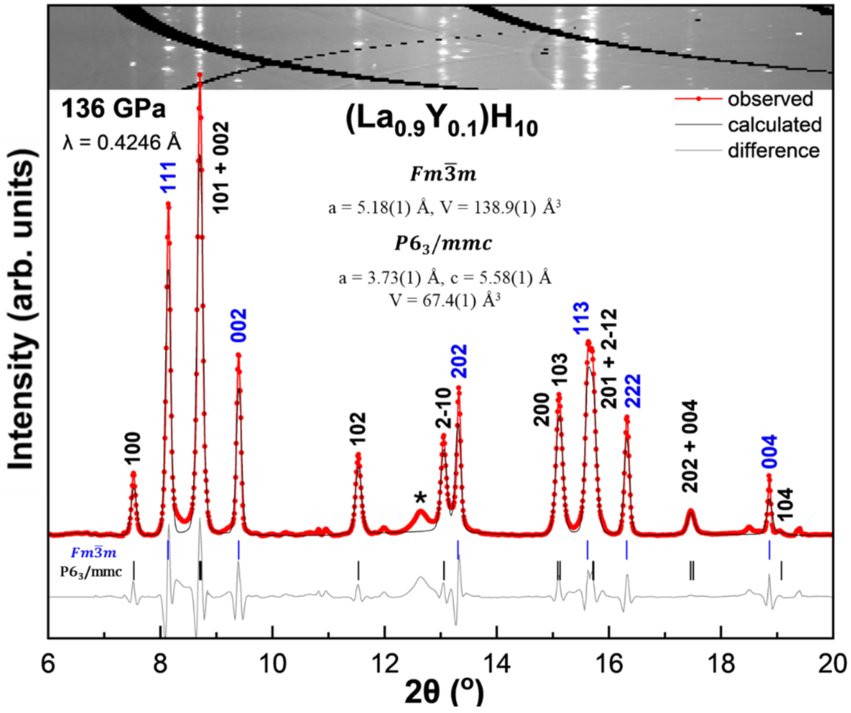

Figure S6: Experimental synchrotron XRD patterns and Le Bail refinements of the $Fm\overline{3}m$ and $P6_3/mmc$ (La,Y)H_{10} phases at 136 GPa. The experimental data, fit, and residuals are shown in red, black, and gray, respectively. Refined lattice parameters for both phases are indicated. Insets show representative 2D diffraction images for reference.

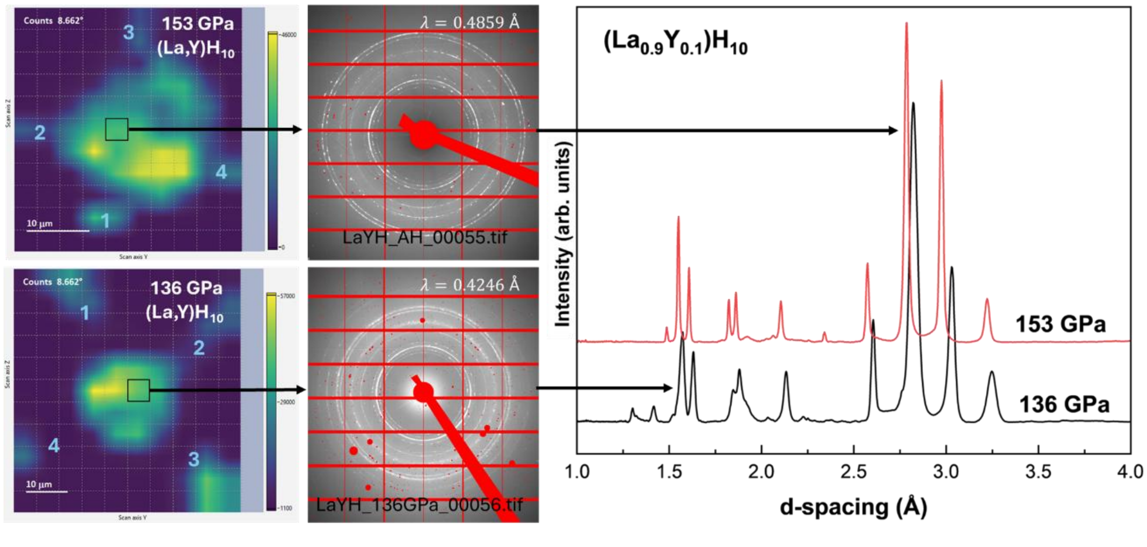

Figure S7: XRD patterns of (La,Y)H_{10} at 153 GPa and 136 GPa in DAC #1, collected from comparable positions in the sample chamber to confirm the presence of cubic and hexagonal phases at both pressures. The data were taken from the central square (55th and 56th positions in a 110-square grid) to ensure consistent sampling. **Left**: XDI maps of the sample chamber. **Middle**: representative 2D diffraction images from the selected positions. **Right**: integrated diffraction patterns in d-spacing, demonstrating that the two datasets are directly comparable.

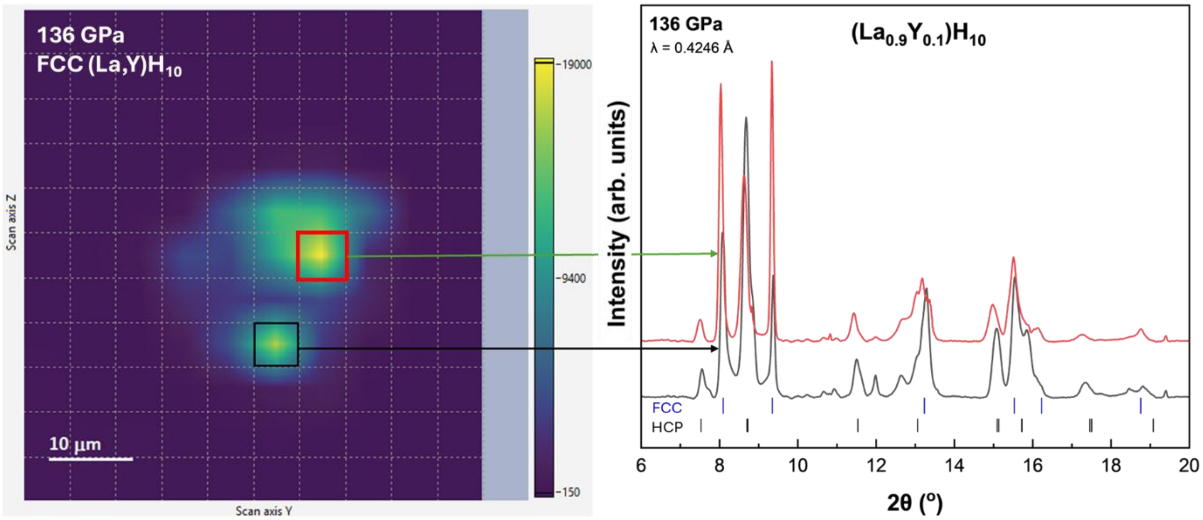

Figure S8: Spatially resolved XRD analysis of the cubic phase at 136 GPa. XDI map of the $Fm\bar{3}m$ (cubic) phase of (La,Y)H_{10} at 136 GPa, showing its spatial distribution across the scanned region. Diffraction patterns extracted from two high-intensity regions of the grid (right) confirm the presence of the cubic phase with slight peak shifts between locations, likely arising from local pressure gradients and lattice relaxation effects.

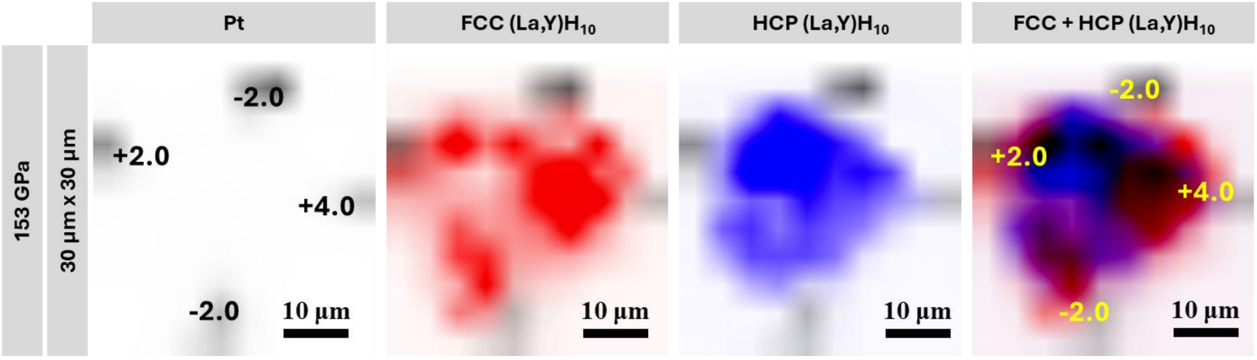

Figure S9: XDI maps with local pressure variation at 153 GPa. A 30 µm × 30 µm raster shows Pt leads (dark gray), cubic $Fm\bar{3}m$ (red), and hexagonal $P6_3/mmc$ (blue). Local pressures at the electrode–sample junctions were derived from Pt(111) using the Pt EOS (K_0 = 266 GPa, K_0' = 5.81, V_0 = 60.3793 Å³ per fcc cell)[6]. The electrodes span 164–170 GPa (ΔP ≈ 6 GPa). ΔP values relative to the median (=166 GPa) are displayed on both the Pt map and the FCC+HCP overlay. Domain boundaries do not align with pressure gradients: FCC and HCP phases coexist across the full ΔP range, indicating pressure is not the primary control on domain arrangement at synthesis conditions. The center diamond-edge Raman measured 153 GPa, slightly lower than Pt-EOS values; such offsets are common above 150 GPa due to scale differences and nonhydrostatic stress[7–10]. For correlation, we emphasize relative ΔP rather than absolute pressure.

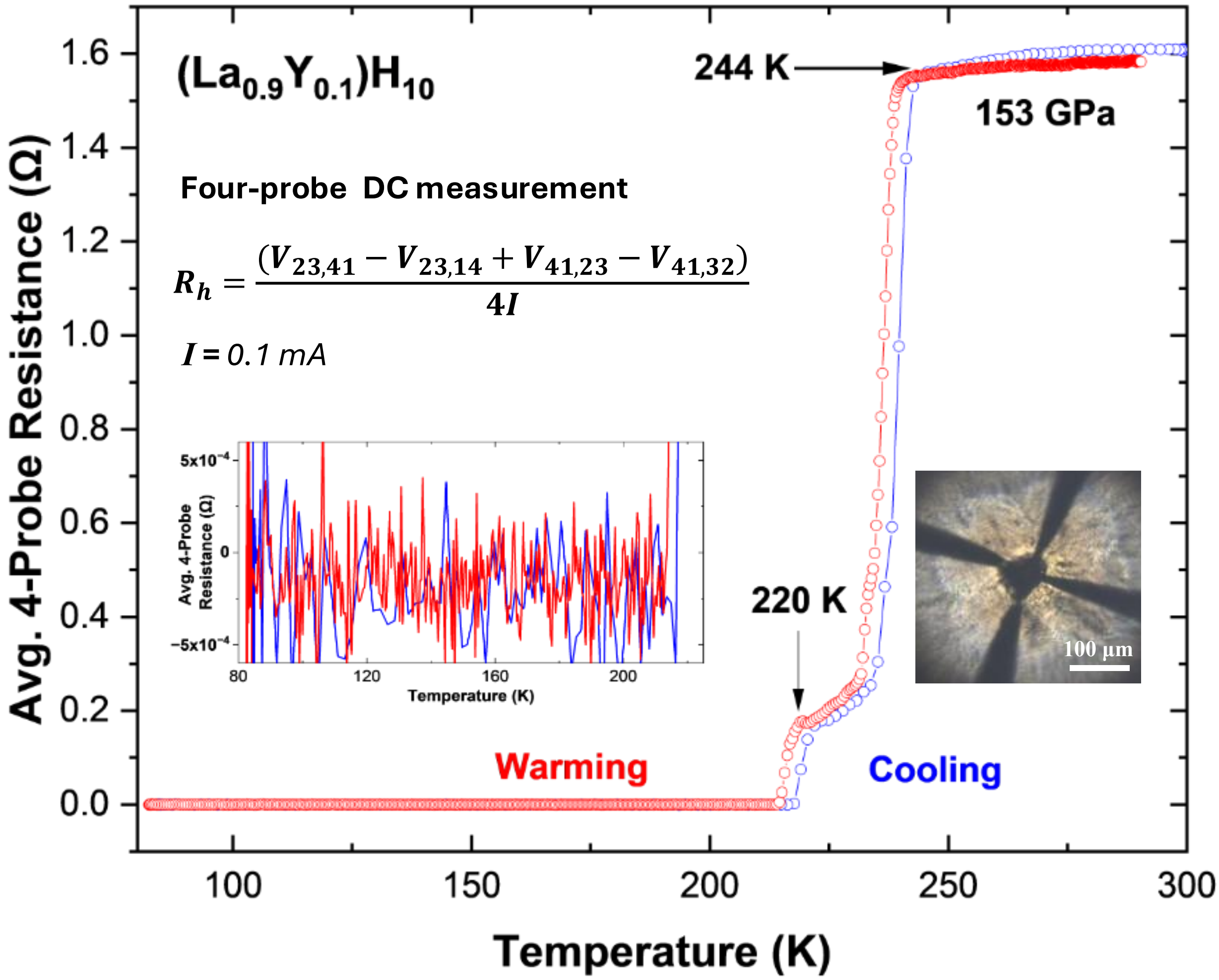

Figure S10: R–T of (La,Y)H_{10} at 153 GPa. Temperature-dependent resistance of $(La_{0.9}Y_{0.1})H_{10}$ measured using a four-probe DC configuration at 153 GPa with an excitation current of 0.1 mA. The plot shows both cooling and warming cycles, revealing two distinct superconducting transitions at approximately 244 K and 220 K. The curves represent the average four-probe resistance, calculated using the expression shown in the inset to eliminate thermoelectric offsets. Insets show a magnified view demonstrating that all signals drop below the instrument's noise floor in the superconducting state, and an optical image of the DAC culet with platinum electrodes.

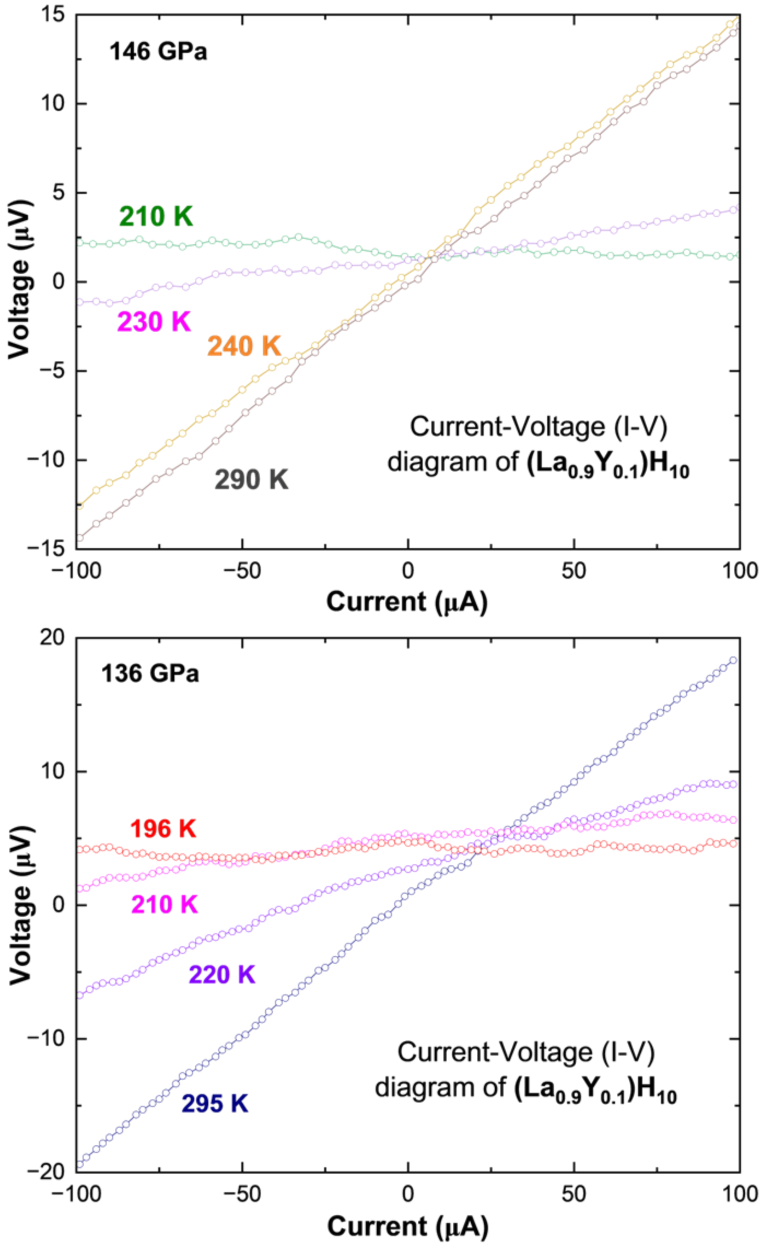

Figure S11: Current–voltage (I–V) characteristics of $(La_{0.9}Y_{0.1})H_{10}$ measured at 146 GPa and 136 GPa across various temperatures, using a delta-mode Keithley 6220 current source and 2182A nanovoltmeter[11]. At 146 GPa, the I–V response is linear at 290 K, consistent with metallic Ohmic behavior. Below ~230 K, the curves become increasingly nonlinear, with a pronounced deviation at 210 K, mirroring the superconducting transition seen in resistance–temperature data. At 136 GPa, similar non-Ohmic behavior emerges below 210 K, indicating persistent superconductivity upon decompression. The nonlinear I–V profiles reflect the formation of a superconducting gap and critical current limitations, consistent with prior high-pressure studies[2]. These measurements provide complementary confirmation of superconductivity in $(La,Y)H_{10}$ and highlight the utility of I–V analysis alongside resistance and structural mapping in mixed-phase superhydrides.

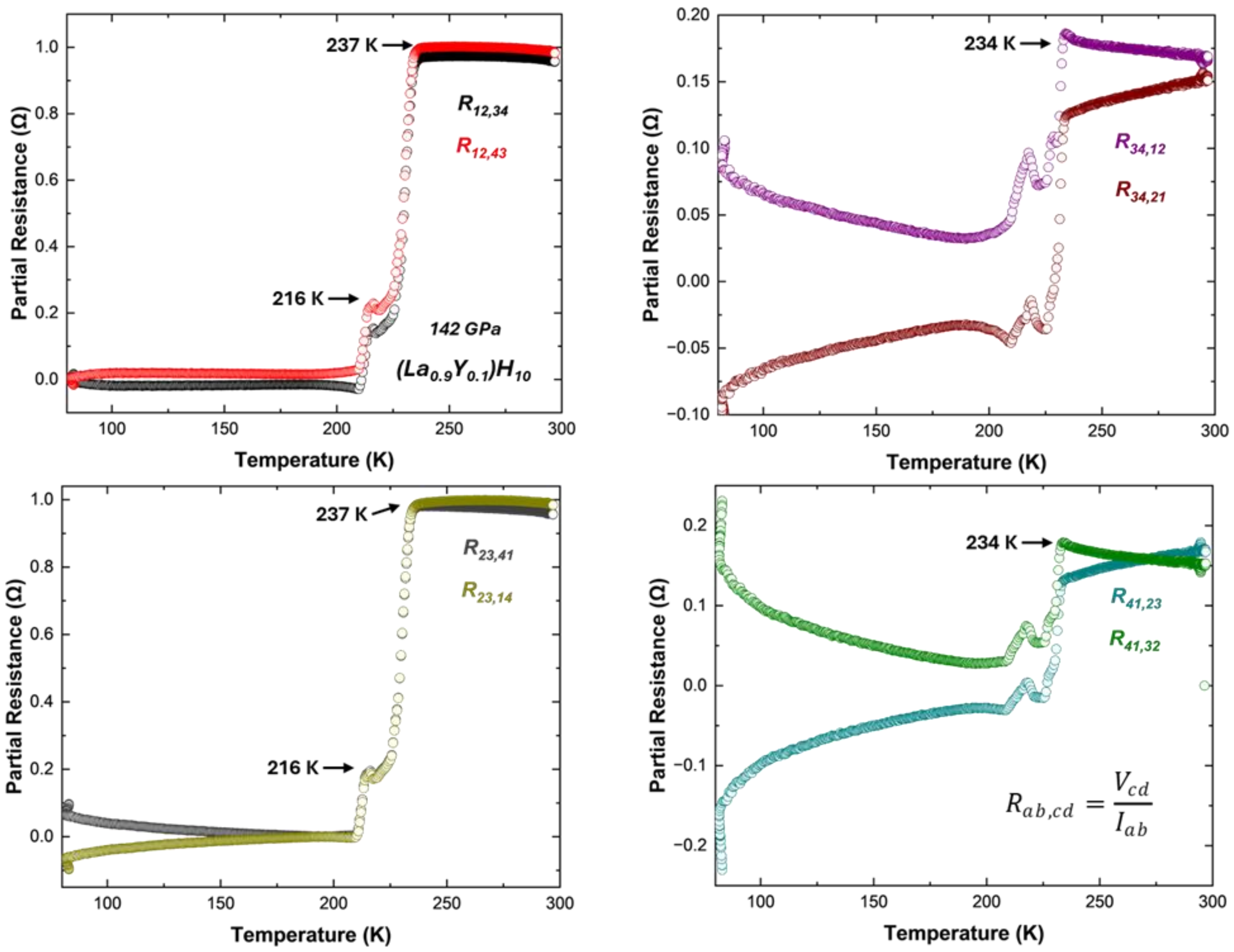

Figure S12: Temperature-dependent four-probe partial resistance traces ($R_{ab,cd}$) of $(La_{0.9}Y_{0.1})H_{10}$ at 142 GPa, measured using eight current–voltage configurations are shown across four panels. Traces corresponding to current paths intersecting FCC-enriched regions (e.g., $R_{34,12}$, $R_{41,23}$) exhibit sharp transitions near 240 K with $\Delta T < 10$ K, while configurations sampling mixed or HCP-rich regions (e.g., $R_{12,34}$, $R_{23,14}$) show broader or multi-step transitions. Negative resistance values below T_c arise from thermoelectric (Seebeck) voltage offsets that dominate the signal after the sample becomes superconducting[11]. These results further support the link between local structural domains and superconducting behavior.

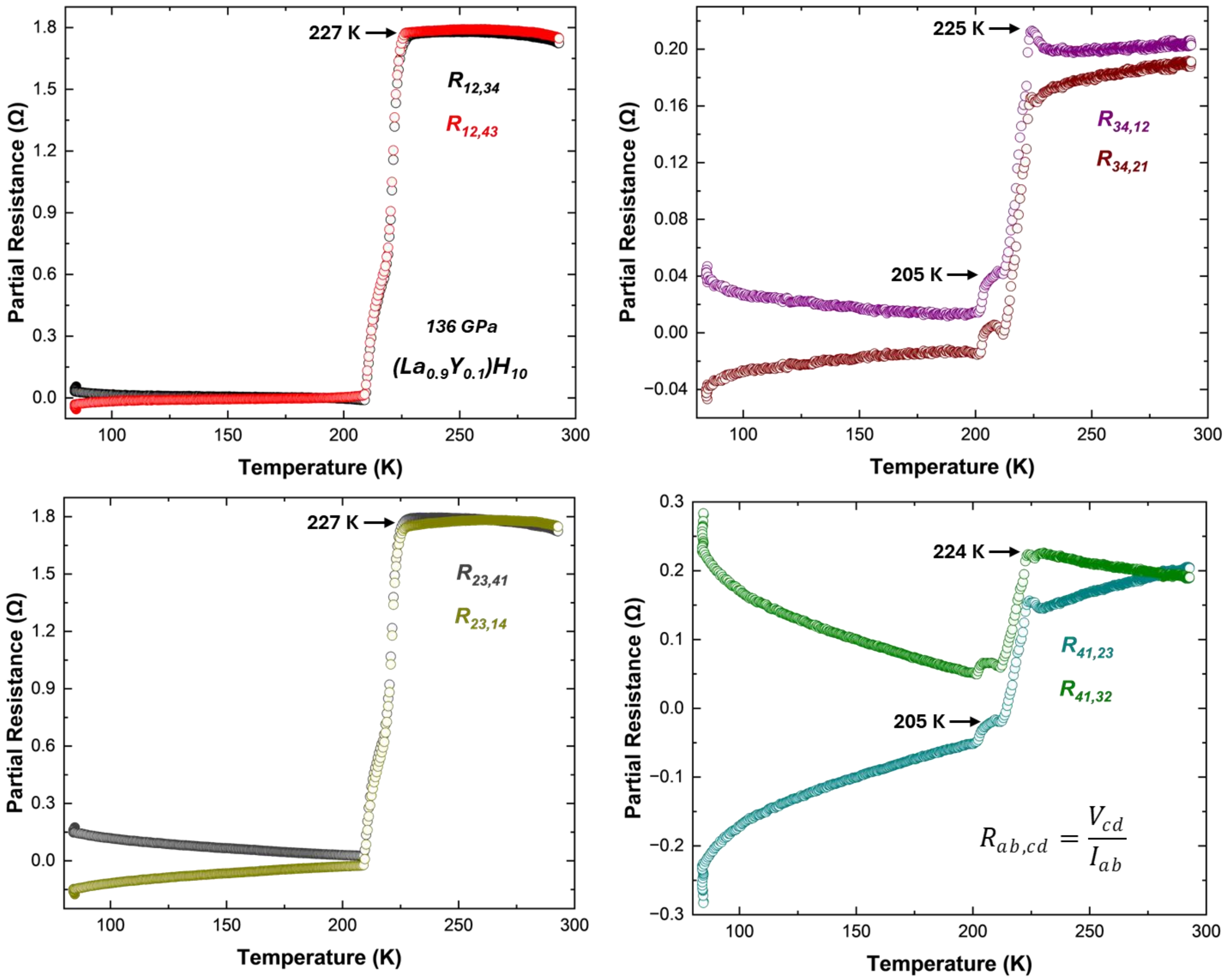

Figure S13: Temperature-dependent four-probe partial resistance traces ($R_{ab,cd}$) of $(La_{0.9}Y_{0.1})H_{10}$ at 136 GPa, measured using eight current–voltage configurations are shown across four panels. Paths intersecting regions with reduced FCC domain (e.g., $R_{12,34}$, $R_{23,41}$) show broader transitions ($\Delta T \approx 20$ K), while those sampling FCC-enriched domains near Pt leads #2 and #3 (e.g., $R_{34,12}$, $R_{41,23}$) exhibit sharp drops near 228 K with secondary features.

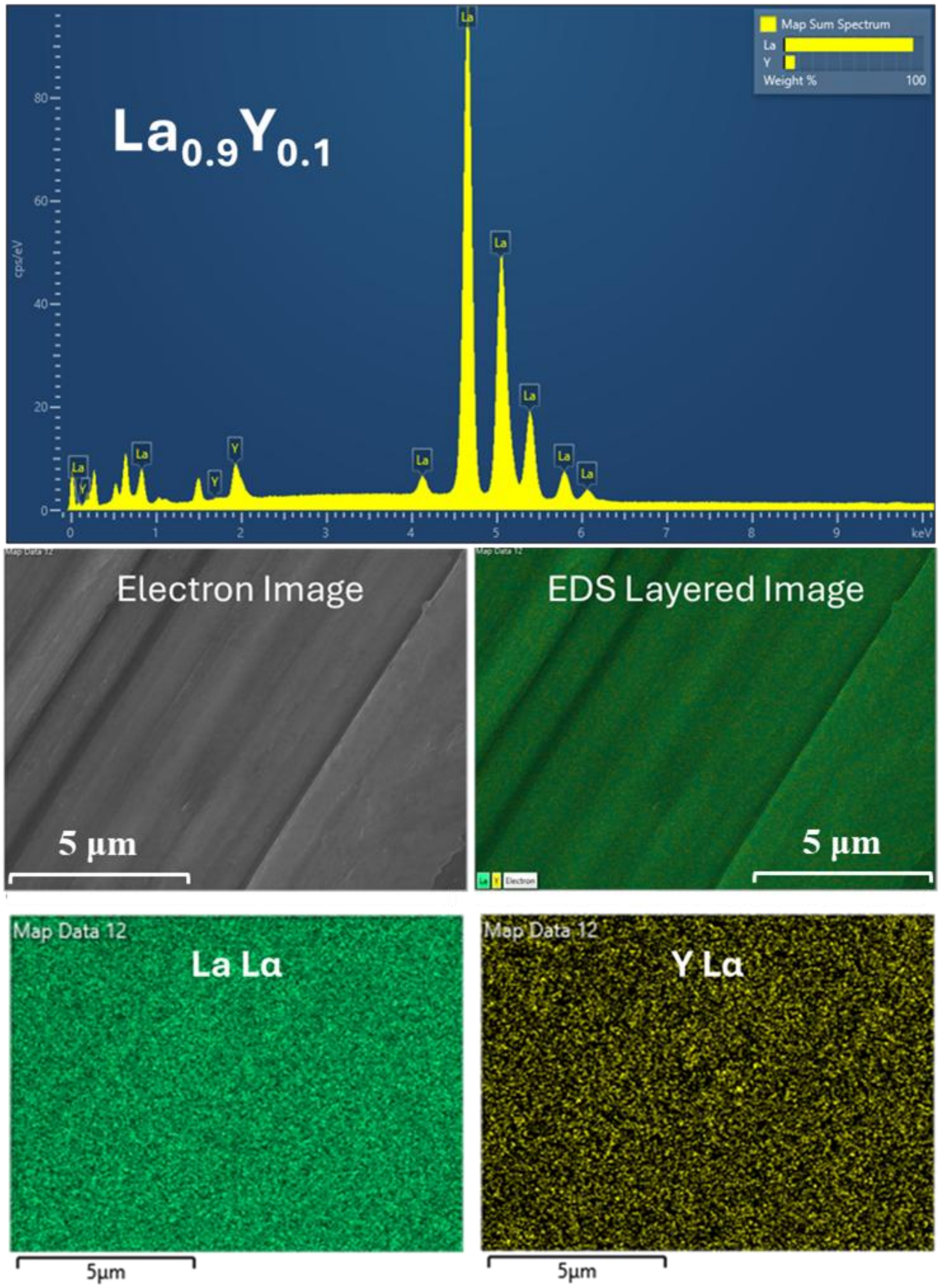

Figure S14: SEM-EDS characterization of the $La_{0.9}Y_{0.1}$ alloy. **Top**: EDS spectrum acquired at 5 µm resolution, showing characteristic peaks of La (Mα at ~0.83 keV, Lα at ~4.65 keV, and Lβ at ~5.48 keV) and Y (Lα at ~1.92 keV). **Middle**: Secondary electron image of the region where EDS data were collected (left), followed by layered EDS images highlighting the distribution of detected elements. **Bottom**: Elemental maps showing the spatial distribution of La (La Lα) and Y (Y Lα), confirming a uniform dispersion of both elements across the analyzed region.

Table S1: Results of SEM–EDS analysis for $La_{0.9}Y_{0.1}$ alloy. The listed values correspond to EDS spectra acquired at 5 μm resolution (Fig. S14). SEM imaging was performed over a 100 μm field of view. Additional EDS measurements at 25 μm and 10 μm gave consistent results, supporting alloy homogeneity across multiple length scales.

$La_{0.9}Y_{0.1}$						
Element	Line Type	Apparent Concentration	k-Ratio	Wt%	Wt% Sigma	Standard Label
Y	L series	1.08	0.01082	7.67	0.15	Y
La	L series	35.12	0.31512	92.33	0.15	LaB_6
Total:				100.00		

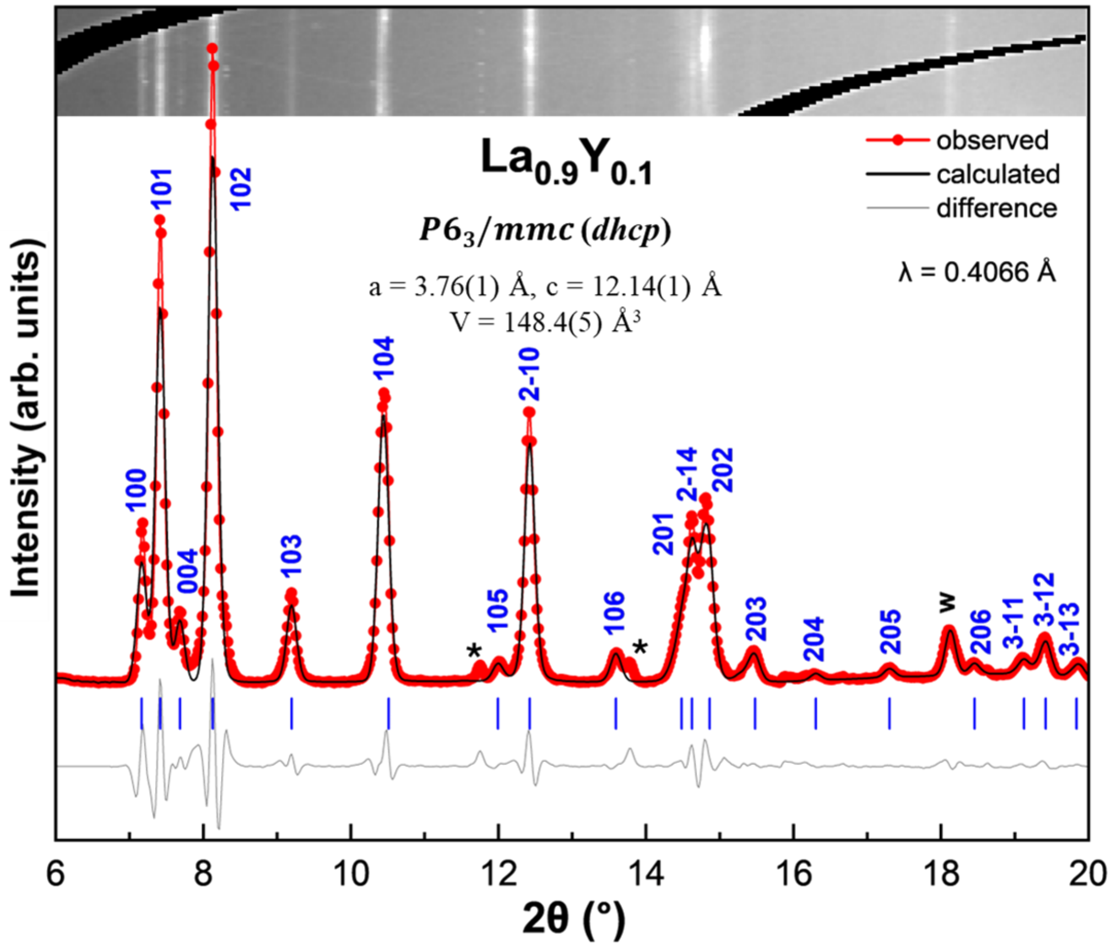

Figure S15: Synchrotron XRD patterns of $La_{0.9}Y_{0.1}$ alloy at ambient conditions. The $La_{0.9}Y_{0.1}$ sample exhibits a dhcp structure. The patterns confirm phase purity and show no evidence of phase separation or residual elemental La or Y, indicating successful alloy formation. * marks unknown peaks.

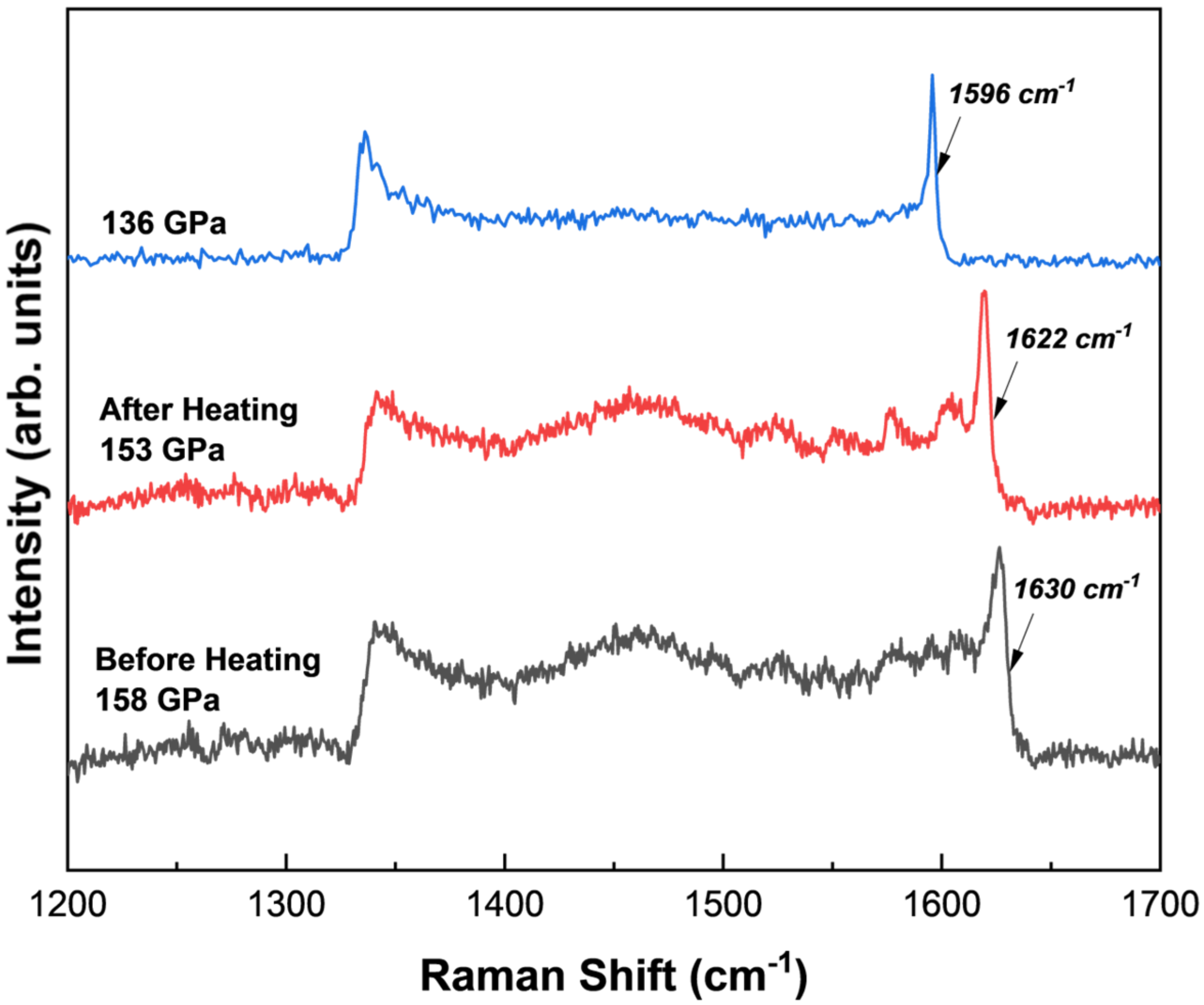

Figure S16: Diamond-edge Raman spectra for pressure calibration (DAC #1). Representative spectra at 158 GPa (before laser heating), 153 GPa (after laser heating), and 136 GPa (during decompression). The diamond edge was measured at the center of the sample.

Note 1: Volume-Based Stoichiometry Analysis of (La,Y)H_{10}

To estimate the hydrogen content of the synthesized clathrate phases, we compared the atomic volumes of the La–Y alloy precursor and the hydrogenated phases, following procedures established for rare-earth hydrides[2,5]. All values reported here were obtained from DAC #1 (Fig. 1). The alloy measurement was performed at 158 GPa, while the hydride phases were measured at 153 GPa; the ±5 GPa difference is within the experimental uncertainty of pressure determination and therefore allows direct comparison.

- $La_{0.9}Y_{0.1}$ alloy precursor ($Fmmm$, 158 GPa):
  Unit cell volume = 58.5 $Å^3$ with 4 metal atoms → atomic volume = 14.63 $Å^3$/atom.
- Cubic $Fm\bar{3}m$ phase (153 GPa):
  Unit cell volume = 136.6 $Å^3$ with 4 formula units → atomic volume = 34.15 $Å^3$/metal atom.
  Expansion relative to alloy = 19.52 $Å^3$/atom.
- Hexagonal $P6_3/mmc$ phase (153 GPa):
  Unit cell volume = 66.1 $Å^3$ with 2 metal atoms → atomic volume = 33.05 $Å^3$/metal atom.
  Expansion relative to alloy = 18.42 $Å^3$/atom.
- Hydrogen reference:
  The atomic volume of hydrogen at 153 GPa is taken as 1.961 $Å^3$/atom, derived from the Vinet EOS of H_2, using parameters: B_0=0.162 GPa, B_0'=6.813, V_0=21.116 $Å^3$ [12].

From these values, the estimated hydrogen content per metal atom is:

$$n_H = \frac{\Delta V}{V_H}$$

- Cubic phase: 19.52 / 1.96 ≈ 9.96 H/metal atom
- Hexagonal phase: 18.42 / 1.96 ≈ 9.34 H/metal atom

These results indicate that both phases are consistent with decahydride stoichiometry, within experimental uncertainty, and support the assignment of the composition as $(La_{0.9}Y_{0.1})H_{10}$. This conclusion is further supported by the pressure–volume trends shown in Figure S4, where the data for both FCC and HCP phases closely follow the reported equation of state of LaH_{10}[1–3,5], consistent with ~10 H per metal atom.

References

1. Geballe, Z. M. *et al.* Synthesis and stability of lanthanum superhydrides. *Angew. Chem. Int. Ed.* **57**, 688–692 (2018).
2. Somayazulu, M. *et al.* Evidence for superconductivity above 260 K in lanthanum superhydride at megabar pressures. *Phys. Rev. Lett.* **122**, 027001 (2019).
3. Drozdov, A. P. *et al.* Superconductivity at 250 K in lanthanum hydride under high pressures. *Nature* **569**, 528–531 (2019).
4. Sun, D. *et al.* High-temperature superconductivity on the verge of a structural instability in lanthanum superhydride. *Nat. Commun.* **12**, 6863 (2021).
5. Semenok, D. V. *et al.* Superconductivity at 253 K in lanthanum–yttrium ternary hydrides. *Mater. Today* **48**, 18–28 (2021).
6. Holmes, N. C., Moriarty, J. A., Gathers, G. R. & Nellis, W. J. The equation of state of platinum to 660 GPa (6.6 Mbar). *J. Appl. Phys.* **66**, 2962–2967 (1989).
7. Akahama, Y. & Kawamura, H. Pressure calibration of diamond anvil Raman gauge to 410 GPa. *J. Phys.: Conf. Ser.* **215**, 012195 (2010).
8. Singh, A. K. & Balasingh, C. The lattice strains in a specimen (hexagonal system) compressed nonhydrostatically in an opposed anvil high pressure setup. *J. Appl. Phys.* **75**, 4956–4962 (1994).
9. Fratanduono, D. E. *et al.* Establishing gold and platinum standards to 1 terapascal using shockless compression. *Science* **372**, 1063–1068 (2021).
10. Yokoo, M. *et al.* Ultrahigh-pressure scales for gold and platinum at pressures up to 550 GPa. *Phys. Rev. B Condens. Matter.* **80**, 104114 (2009).
11. Keithley, J. F. & Inc, K. I. *Low Level Measurements Handbook: Precision DC Current, Voltage, and Resistance Measurements*. (Keithley Instruments, 2004).
12. Loubeyre, P. *et al.* X-ray diffraction and equation of state of hydrogen at megabar pressures. *Nature* **383**, 702–704 (1996).
